# C-Reactive Protein/Albumin Ratio Predicts 90-Day Mortality of Septic Patients

**DOI:** 10.1371/journal.pone.0059321

**Published:** 2013-03-12

**Authors:** Otavio T. Ranzani, Fernando Godinho Zampieri, Daniel Neves Forte, Luciano Cesar Pontes Azevedo, Marcelo Park

**Affiliations:** 1 Intensive Care Unit, Emergency Medicine Discipline, Hospital das Clínicas, Universidade de São Paulo, São Paulo, Brazil; 2 Intensive Care Unit, Hospital Alemão Oswaldo Cruz, São Paulo, Brazil; 3 Research and Education Institute, Hospital Sírio-Libanês, São Paulo, Brazil; D'or Institute of Research and Education, Brazil

## Abstract

**Introduction:**

Residual inflammation at ICU discharge may have impact upon long-term mortality. However, the significance of ongoing inflammation on mortality after ICU discharge is poorly described. C-reactive protein (CRP) and albumin are measured frequently in the ICU and exhibit opposing patterns during inflammation. Since infection is a potent trigger of inflammation, we hypothesized that CRP levels at discharge would correlate with long-term mortality in septic patients and that the CRP/albumin ratio would be a better marker of prognosis than CRP alone.

**Methods:**

We evaluated 334 patients admitted to the ICU as a result of severe sepsis or septic shock who were discharged alive after a minimum of 72 hours in the ICU. We evaluated the performance of both CRP and CRP/albumin to predict mortality at 90 days after ICU discharge. Two multivariate logistic models were generated based on measurements at discharge: one model included CRP (Model-CRP), and the other included the CRP/albumin ratio (Model-CRP/albumin).

**Results:**

There were 229 (67%) and 111 (33%) patients with severe sepsis and septic shock, respectively. During the 90 days of follow-up, 73 (22%) patients died. CRP/albumin ratios at admission and at discharge were associated with a poor outcome and showed greater accuracy than CRP alone at these time points (p = 0.0455 and p = 0.0438, respectively). CRP levels and the CRP/albumin ratio were independent predictors of mortality at 90 days (Model-CRP: adjusted OR 2.34, 95% CI 1.14–4.83, p = 0.021; Model-CRP/albumin: adjusted OR 2.18, 95% CI 1.10–4.67, p = 0.035). Both models showed similar accuracy (p = 0.2483). However, Model-CRP was not calibrated.

**Conclusions:**

Residual inflammation at ICU discharge assessed using the CRP/albumin ratio is an independent risk factor for mortality at 90 days in septic patients. The use of the CRP/albumin ratio as a long-term marker of prognosis provides more consistent results than standard CRP values alone.

## Introduction

Sepsis is a major cause intensive care unit (ICU) admission and is associated with high morbidity and mortality rates [1], [2]. In addition to its clear impact on short-term mortality and morbidity, the impact of sepsis on the individual persists after the period of critical illness, and increased mortality after ICU discharge is reported frequently [3]–[6]. Moreover, the inflammation present during the stay in the ICU may cause subsequent deleterious effects [7]. For example, patients who survive sepsis due to pneumonia are more at risk for cardiovascular disease (including stroke and myocardial infarction) after discharge from the hospital, suggesting that an acute episode of systemic inflammation has a long-lasting effect [4].

C-reactive protein (CRP) is an acute-phase protein that has been evaluated extensively in the critical setting [8]. Authors have suggested that CRP can be used both as a diagnostic tool for sepsis and as a guide when evaluating treatment efficacy in infected patients [9], [10]. Measurement of CRP could also aid in the decision-making process for ICU discharge [11]. Moreover, CRP levels correlate with the degree of inflammation during the early course of illness [12]. Although some studies showed that CRP levels upon discharge from the ICU could be a reliable marker of outcomes during the post-ICU period, no studies have focused on septic patients, and few have evaluated the long-term prognosis [13]. Most studies have focused on readmission to the ICU or mortality in the hospital [14]–[17]. In addition to CRP, serum albumin may be an important short- and long-term marker for prognosis [18]–[20]. Serum albumin is a negative acute-phase protein; thus, the degree of hypoalbuminemia in critically ill patients correlates with the intensity of the inflammatory response triggered by infection [5], [15], [19]. Therefore, CRP and serum albumin levels should diverge during sepsis. The use of a ratio between CRP and albumin would provide a variable capable of merging the information provided by CRP and albumin into an index that correlated positively with infection, i.e., a higher ratio indicates higher inflammatory status [21].

Because infection is one of the strongest stimuli of the inflammatory response, we hypothesized that CRP levels at ICU discharge would be an important marker of long-term mortality in patients with sepsis. In addition, we investigated whether merging information from albumin with CRP via a CRP/albumin ratio [21] would result in a marker of mortality with improved consistency compared with CRP alone.

## Materials and Methods

### Patients

This study is a retrospective analysis of prospectively collected data from 1210 consecutive critically ill patients who were admitted to the medical ICU of the Emergency Department of the Hospital das Clínicas of São Paulo in Brazil. Data were retrieved between January 2005 and July 2009. Hypotheses were generated before data analysis and after data collection.

This study protocol followed the statements of the Declaration of Helsinki. The institutional review board (IRB), called the *Comissão para Análise de Projetos de Pesquisa* (CAPPesq), reviewed and approved this study (CAPPesq – protocol number 9151/12). The requirement for written, informed consent was waived because there was no intervention, and only a database that had guaranteed confidentiality was used.

We included patients that were discharged alive from the ICU following a stay of at least 72 hours (ICU length-of-stay, ICU LOS). For patients with multiple ICU admissions, the first admission only was recorded. Patients with major pieces of data missing or patients transferred to other ICUs were excluded.

### Data collection

Data extracted from our electronic database included demographics (age, gender) and comorbidity profile, syndrome at admission, source of admission, type of admission, severity of illness (Acute Physiology and Chronic Health Evaluation II-APACHE II and Sequential Organ Failure Assessment – SOFA [22]), mechanical ventilation (MV), renal replacement therapy (RRT) and vasoactive drug used. We also recorded the hour of discharge from the ICU, the LOS in hospital prior to ICU admission and the LOS during the ICU period. Follow-up was evaluated at 90 days after ICU discharge or upon death. Mortality was assessed via the hospital records. Any unscheduled ICU readmission in the same hospitalization after discharge from our unit was recorded.

### Laboratory data

Blood samples were collected daily according to our ICU standard of care and analyzed in the central laboratory of our institution. We collected the following laboratory variables at ICU discharge: arterial blood gas, lactate, albumin, hemoglobin levels, white blood cell (WBC) and platelet counts and CRP. CRP measurements were performed using an immunoturbidimetric method that employed a commercially available test (BIOTÉCNICA Indústria e Comércio Ltda., Minas Gerais, Brazil) where normal values were <5 mg/L. We evaluated CRP levels at ICU admission, the maximum value during the ICU stay and in the last 48 and 24 hours prior to ICU discharge. CRP values were only included in the analysis if the sample was taken within a predefined time window to avoid bias due to CRP half-life and the sample collection routine [11].

### Albumin

Serum albumin was collected on a daily basis. If albumin levels at discharge were not available, we included values that were collected during the last 48 hours prior to discharge. In our service, albumin is not available to resuscitate septic patients.

The ICU discharge time was collected from the hospital's official electronic system. All laboratory data were available to the clinicians prior to making the decision to discharge each patient.

### Statistical Analysis

Data are presented as the mean ± standard deviation (SD) or the median and the 25^th^ and 75^th^ percentiles (IQR) if distributions were normal or skewed, respectively. Baseline characteristics of the post-ICU survivors and non-survivors were compared using Mann-Whitney or unpaired *t-*tests, as appropriate. Fisher's exact test or Chi-squared tests were used for dichotomous variables.

Sensitivity, specificity, positive and negative predictive values and diagnostic odds ratios were calculated using previously described methods [23].

Two different logistic regression models were developed to evaluate the prognostic factors for mortality at 90 days after ICU discharge. Model-CRP included CRP at discharge whereas Model-CRP/albumin included the CRP/albumin ratio. All independent variables with P<0.25 [24] on the univariate analysis were included in the initial model and were then selected using a likelihood-ratio backward elimination method. One variable was removed at a time if it did not contribute to the model, which was assessed using the likelihood ratio test (P<0.050). Continuous variables were checked for the assumption of linearity in the logit. If this assumption was not proven, the variable was categorized. Single collinearity was evaluated using Pearson`s correlation for independent variables, and multicollinearity was evaluated using the variance inflation factor (VIF). The odds ratio (OR) and corresponding 95% confidence limits (CI) for each variable were computed. The ability of the models to predict the patient outcomes was assessed using the area under the receiver operating characteristic (ROC). Estimated area-under-the-curve (AUC) values were compared using the nonparametric method described by Hanley and NcNeil [25]. The calibration ability of each model was evaluated using the Hosmer-Lemeshow goodness-of-fit test [24]. This test compares the number of observed deaths with the number of predicted deaths in the risk groups for the entire range of death probabilities. Good calibration (a nonsignificant P value) suggests that the predicted probabilities are similar to the observed results. The cut-off values showing the highest accuracy were determined using the sensitivity/specificity versus criterion value plot. To explore the role of the biomarkers on the 90-day-mortality prediction, we retrieved the predicted 90-day risk of mortality for each patient from the final model, with and without the biomarker, and plotted it against the respective absolute value of age and SOFA score.

Statistical significance was set to P<0.05 (two-tailed). All statistical tests were performed using the commercial SPSS19.0 package for Windows (Chicago, Illinois, USA) and MedCalc Statistical Software (Mariakerke, Belgium).

## Results

**Figure 1:**
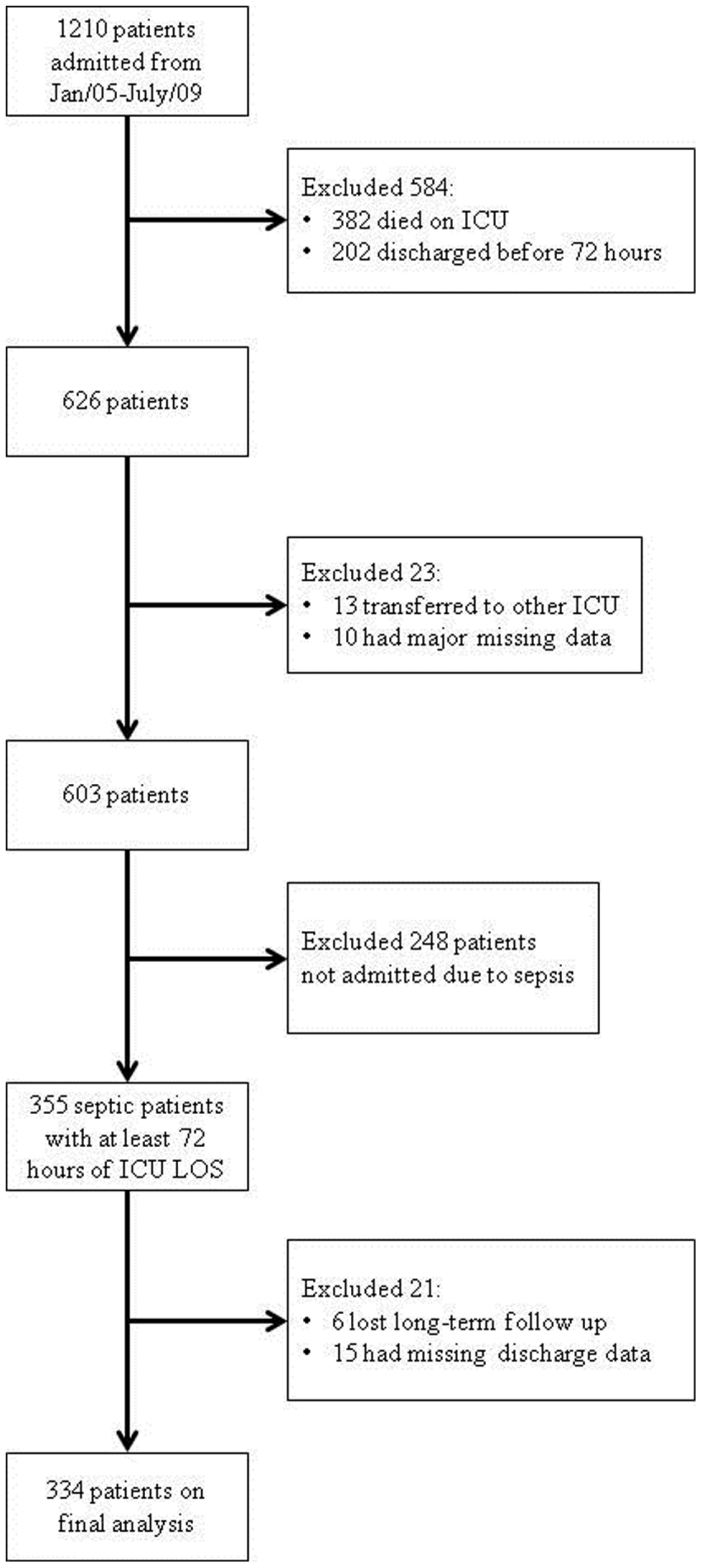
Study flowchart.

Of the initial sample of 1210 patients, 382 patients died in the ICU, 202 patients were discharged within 72 hours of ICU admission, 13 patients were transferred to another ICU and 10 had major missing data. Of the remaining 603 patients, 248 patients without sepsis were admitted. Twenty-one patients were further excluded because of missing discharge CRP and/or albumin data (15 patients) and loss during long-term follow-up (six patients). Three hundred and thirty four patients remained for the final analysis. The study flowchart is shown in figure 1. ICU mortality for septic patients who stayed in the ICU for at least 72 hours was 43%.

Baseline features that were associated with 90-day mortality based on the univariate analysis were age, APACHE II score, SOFA at admission and at discharge, origin of patients (ward and emergency room) and most of the comorbidities (Table 1).

**Table 1:** Baseline features associated with 90-day mortality.

	Alive (n = 261)	Death (n = 73)	p value^a^
**Characteristics**			
Age – yo, SD	48±18	60±19	<0.001
Male Gender, n (%)	148 (57)	39 (53)	0.62
APACHE^b^ II score, median [IQ]	16 [10]–[21]	19 [14]–[26]	<0.001
SOFA^c^ at admission, median [IQ]	5 [3]–[8]	7 [5]–[10]	<0.001
SOFA^c^ at discharge, median [IQ]	2 [1]–[3]	3 [2]–[5]	<0.001
**Type of admission,** n (%)			
Medical	223 (85)	29 (81)	0.34
**Origin of Patients,** n (%)			0.078
Ward	87 (33)	37 (51)	0.007
Emergency Room	156 (60)	31 (43)	0.008
Other ICU	10 (4)	2 (3)	1.00
Operating-room	2 (1)	1 (1)	0.52
Step-down unit	3 (1)	2 (3)	0.30
Other	3 (1)	-	-
**Comorbidites, n [IQ]**	1 [0–2]	2 [1]–[3]	<0.001
Hypertension, n (%)	113 (43)	47 (64)	0.001
Diabetes mellitus, n (%)	44 (17)	20 (27)	0.043
Chronic renal failure, n (%)	35 (13)	20 (27)	0.004
Chronic heart failure, n (%)	29 (11)	18 (25)	0.003
Chronic coronary disease, n (%)	18 (7)	18 (25)	<0.001
Chronic obstructive pulmonary disease, n (%)	16 (6)	14 (19)	0.001
Chronic liver disease, n (%)	8 (3)	1 (1)	0.69
AIDS^d^, n (%)	15 (6)	-	-
Cancer, n (%)	25 (10)	13 (18)	0.050

a – p value related to comparison between alive and dead patients; b – Acute Physiology and Chronic Health Evaluation; c – Sequential Organ Failure Assessment; d – acquired immunodeficiency syndrome.

With regard to septic status at ICU admission, there were 229 (67%) and 111 (33%) patients with severe sepsis and septic shock, respectively. The most common areas of infection were lungs (164, 49%) followed by abdominal tissue (48, 14%) and soft tissue (34, 10%). There was no difference in area infected between survivors and non-survivors (p = 0.169). The need for MV, RRT and use of vasoactive drugs were more frequent in patients who died during the 90 days after discharge (Table 2). Neither night-time discharge (21% versus 23%, p = 0.66) nor weekend discharge was not associated with 90-day mortality (19% versus 23%, p = 0.52). Hospital LOS prior to ICU admission was longer in non-survivors compared with survivors (4 [2]–[14] days versus 1 [0–6] day, p<0.01 and 8 [5]–[17] days versus 7 [4]–[11] days, p = 0.016, respectively).

**Table 2:** Organ support during ICU stay and Laboratorial data at ICU discharge related to 90-days outcome.

	Alive (n = 261)	Death (n = 73)	p value^a^
**Support During ICU Stay,** n (%)			
Mechanical Ventilation	135 (52)	49 (67)	0.019
Renal Replacement Therapy	35 (13)	26 (36)	<0.001
Vasoactive Drugs	130 (50)	47 (64)	0.027
**Laboratorial data at ICU discharge,** median [IQR)			
Hemoglobin (g/dL)	9.3 [8.1–11.2]	8.6 [7.8–9.6]	0.001
WBC^b^	9,520 [6,630–13,005]	10,650 [6,995–14,385]	0.152
Platelets	252 [166–368]	198 [124–333]	0.009
Creatinine (mg/dL)	0.80 [0,60–1,20]	1.10 [0,72–2,11]	<0.001
Albumin at admission (g/L)	25 [21]–[31]	23 [20]–[27]	0.001
Albumin at discharge (g/L)	25 [22]–[29]	22 [19]–[27]	<0.001
CRP^c^ admission (mg/L)	162 [75–263]	200 [118–325]	0.024
CRP^c^ maximum (mg/L)	186 [131–289]	206 [158–357]	0.042
CRP^c^ at discharge (mg/L)	47 [25–98]	77 [39–122]	0.022
CRP^c^/albumin ratio admission	6.2 [3.0–11.2]	8.9 [5.1–14.6]	0.005
CRP^c^/albumin ratio at discharge	1.9 [0.9–4.3]	3.2 [1.6–5.5]	0.005

a – p value related to comparison between alive and dead patients; b – white blood cell; c – C-reactive protein.

Laboratory features associated with the 90-day mortality included hemoglobin levels, platelet counts, serum creatinine, albumin levels at admission and discharge, CRP levels upon admission and discharge and maximum CRP during the ICU stay (Table 2). Univariate analysis showed that CRP/albumin ratios at admission and at discharge were associated with death during the 90 days of follow-up (Table 2).

**Figure 2:**
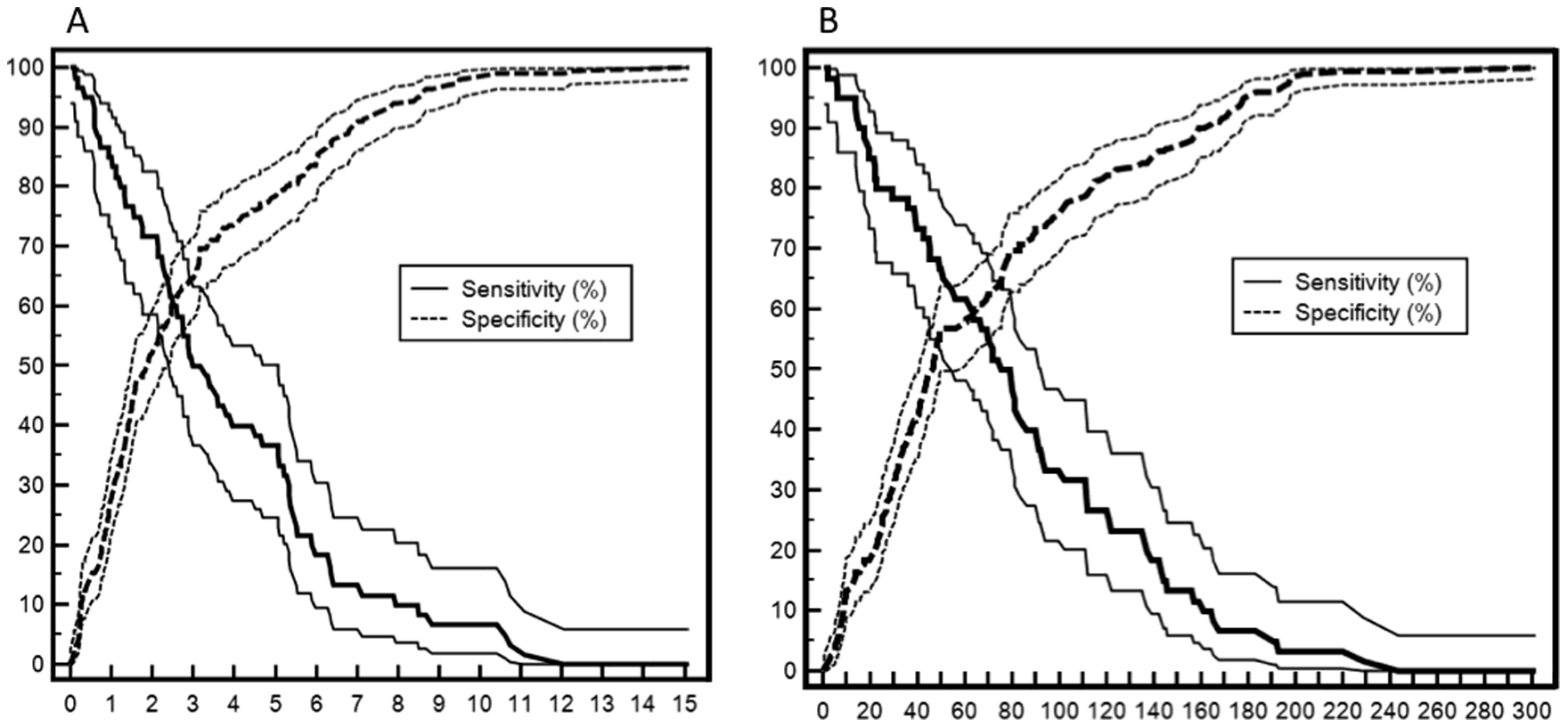
Plot versus criterion value curves for the CRP/albumin ratio and mortality at 90 days (Panel A) and for CRP versus 90-day mortality (Panel B). The X-axis shows the CRP/albumin ratio (Panel A) and serum CRP levels in mg/L (Panel B). The Y-axis shows the percentage. The solid and dashed lines indicate sensitivity and specificity with 95% confidence intervals, respectively.

**Figure 3:**
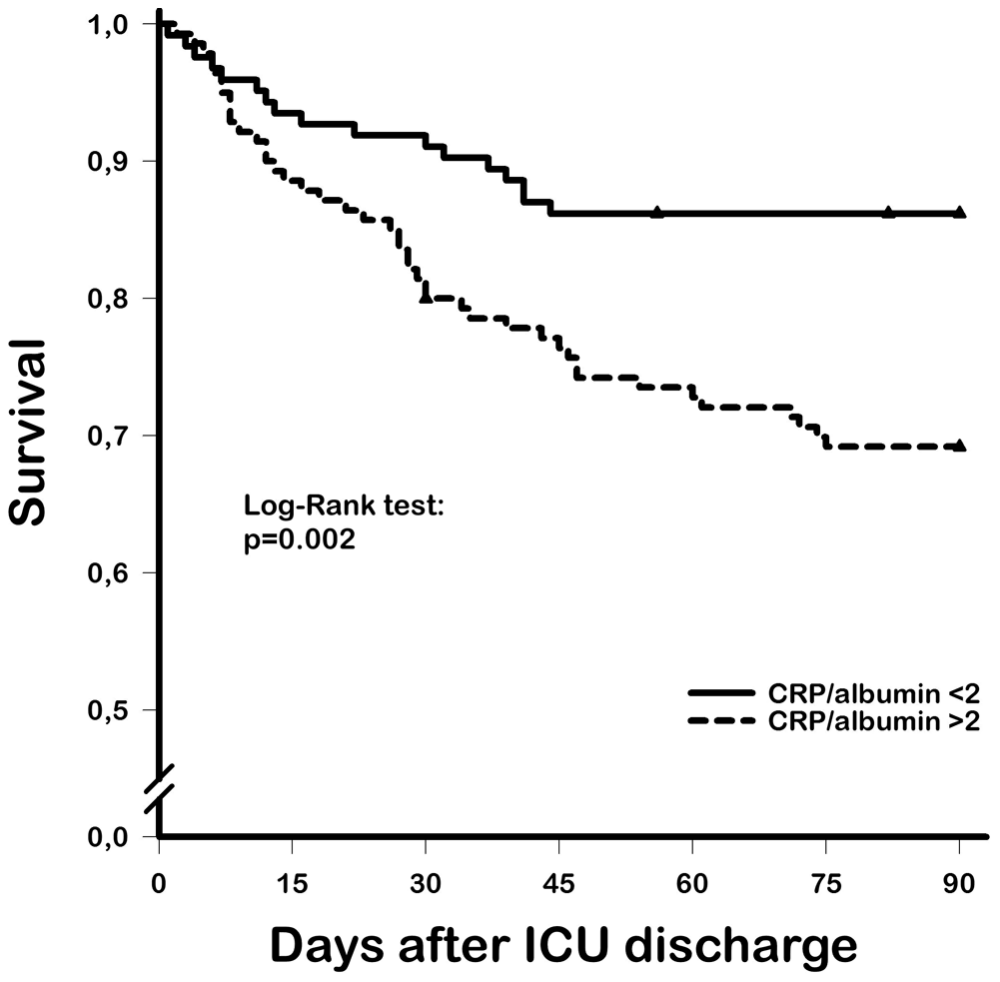
Kaplan-Meier curves showing the 90-day survival of septic patients after discharge from the intensive care unit. *Solid line*, CRP/albumin ratio <2 group; *dashed line*, CRP/albumin ratio >2 group; *triangles*, censored patients.

The abilities of the cutoff, AUC, sensibility, specificity, positive and negative predictive values, diagnostic odds ratio and accuracy of CRP, albumin and CRP/albumin ratio (both at admission and at discharge) to predict death at up to 90 days after ICU discharge are shown in table 3. The CRP/albumin ratio at discharge showed the best AUC and greatest sensibility. The CRP/albumin ratio at ICU admission and discharge showed greater AUC than CRP alone at these time points (p = 0.0455 and p = 0.0438, respectively). The optimal cut-off was 50 mg/L for CRP at ICU discharge and 2 for the CRP/albumin ratio (Figure 2). The 90-day survival is depicted in figure 3, which shows lower survival in patients with CRP/albumin ratios >2 (log-rank test: p = 0.002).

**Table 3:** Evaluation of potentially useful markers of 90-day mortality after discharge.

	AUC (95% CI)	P	Cutt-off	Sensibility	Specificity	PPV^a^	NPV^b^	LR+^c^	LR−^d^	DOR^e^	Accuracy
CRP^f^ at admission	0.590 (0.511–0.670)	0.024	196	52	67	31	83	1.58	0.72	2.20	64
Albumin at admission	0.621 (0.545–0.697)	0.001	25	65	51	29	83	1.33	0.69	1.93	54
CRP^f^/albumin at admission	0.612 (0.534–0.689)	0.005	8.7	54	66	31	83	1.59	0.70	2.28	63
CRP^f^ at discharge	0.597 (0.517–0.678)	0.022	50	68	55	31	85	1.51	0.58	2.60	58
Albumin at discharge	0.647 (0.572–0.722)	<0.001	25	65	51	29	83	1.33	0.54	2.79	55
CRP^f^/albumin at discharge	0.619 (0.540–0.698)	0.005	2	72	52	31	86	1.50	0.54	2.79	57

a – PPV  =  positive predictive value; b – NPV  =  negative predictive value; c- LR+  =  Likelihood ratio for a positive result; d – LR−  =  Likelihood ratio for a negative result; e – DOR  =  diagnostic odds ratio (calculated dividing LR+ by LR-); f – CRP  =  C-reactive protein;

Comparisons between AUCs: CRP/Albumin at admission vs. CRP at admission: p = 0.0455; CRP/Albumin at discharge vs. CRP at discharge: p = 0.0438. For all other comparisons: p = NS.

We also created two models to predict the 90-day mortality. The final multivariate analysis after the selection procedure included age, SOFA score at discharge, chronic comorbidities, hemoglobin levels at discharge, ICU LOS (divided into tertiles) and CRP at discharge (Model-CRP) or CRP/albumin ratio (Model-CRP/albumin) (Table 4). CRP levels at admission, maximum CRP values and CRP levels at 48 h prior to discharge did not remain in the final model. The discrimination of both models was good: Model-CRP showed an AUC of 0.810 (0.755–0.855, p<0.001), and Model-CRP/albumin showed an AUC of 0.815 (0.762–0.860, p<0.001). Both models showed similar discriminative capability (Hanley and McNeil test; p = 0.2483). However, the Model-CRP/albumin was better calibrated than the Model-CRP (Hosmer-Lemeshow goodness-of-fit test: p = 0.100 for Model-CRP/albumin and p = 0.0007 for Model-CRP). This latter result implies that the observed and predicted mortalities for Model-CRP were significantly different.

**Table 4:** Multivariate models to predict 90-day mortality after ICU discharge.

Model-CRP^a^	OR (95% CI)	P	Model-CRP^a^/albumin	OR (95% CI)	P
Age*	1.34 (1.08–1.67)	0.009	Age*	1.32 (1.07–1.65)	0.011
SOFA^b^ at discharge^#^	1.35 (1.14–1.60)	0.001	SOFA^b^ at discharge^#^	1.36 (1.15–1.61)	<0.001
Chronic comorbidities	1.44 (1.10–1.90)	0.010	Chronic comorbidities	1.40 (1.06–1.83)	0.018
Hemoglobin at discharge^#^	0.79 (0.65–0.96)	0.015	Hemoglobin at discharge^#^	0.80 (0.66–0.96)	0.019
ICU LOS^c^, day		0.035	ICU LOS^c^, day		0.035
3–7	Reference		3–7	Reference	
7–14	1.04 (0.43–2.51)	0.92	7–14	1.00 (0.41–2.34)	0.96
>14	2.88 (1.23–6.73)	0.015	>14	2.83 (1.21–6.60)	0.016
CRP^a^ at discharge >50 mg/dL	2.34 (1.14–4.83)	0.021	CRP^a^/albumin ratio at discharge >2	2.18 (1.10–4.67)	0.035

* OR calculated per 10 units increase; # OR calculated per 01 unit increase; a – CRP: C-reactive protein; b – SOFA: sequential organ failure assessment; c – length-of-stay.

**Figure 4:**
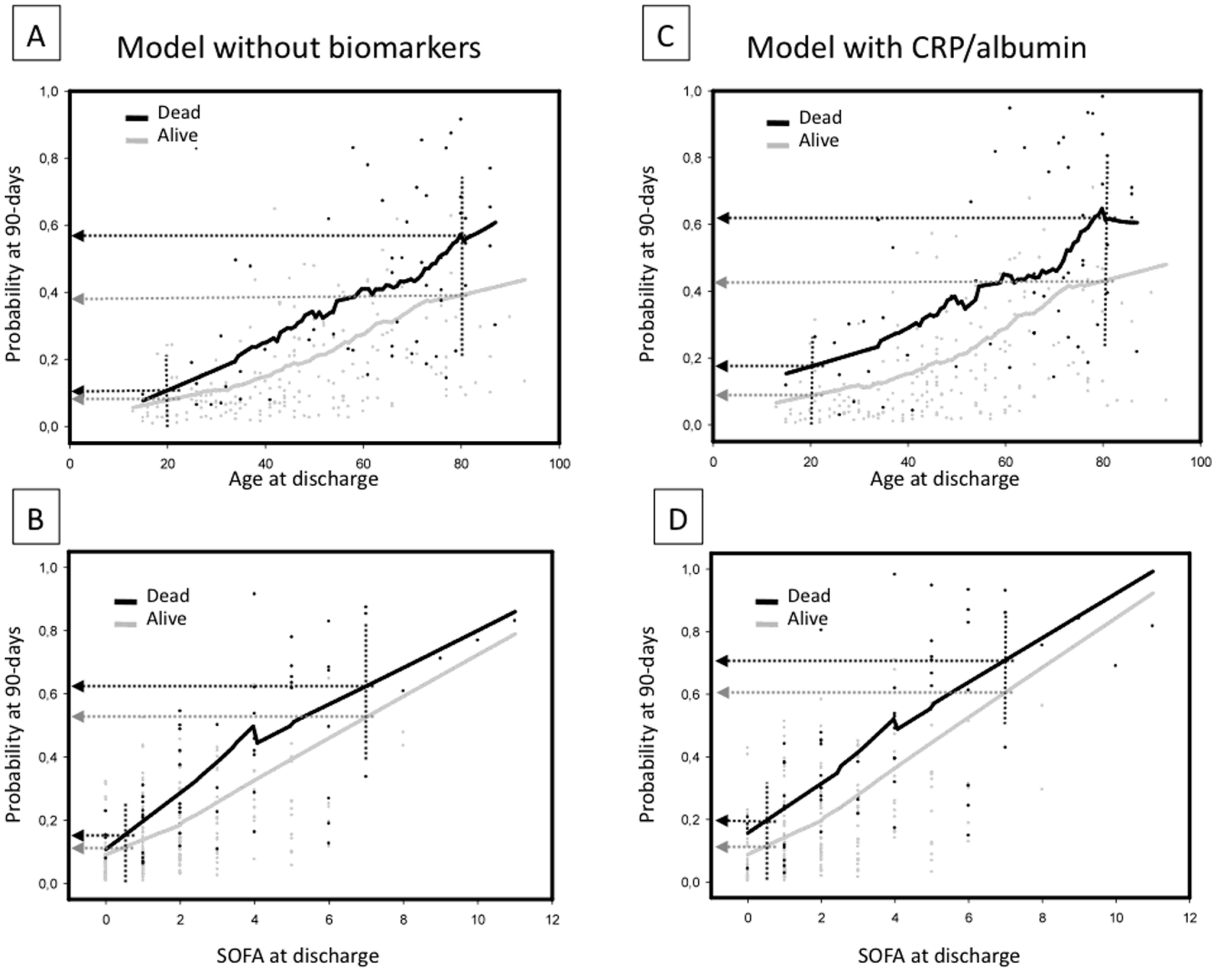
Model of the probability of mortality at 90 days after ICU discharge. The probability of death at 90 days after ICU discharge is depicted using a multivariate logistic model. In A and C, the model is depicted versus age and in B and D versus SOFA score. The models A and B include age, SOFA score, chronic comorbidities, hemoglobin levels and ICU LOS. The models C and D include all variables cited above in addition to the CRP/albumin ratio. The black and gray solid lines indicate the calculated probability of death for dead and alive patients, respectively. For young and less severe patients, the model CRP/albumin ratio (C and D) could differentiate better between survivors and non-survivors than the model without biomarkers. However, as age and SOFA score increase, both models presented similar differences between survivors and non-survivors. The lines were fitted using a loess smooth function.

Using the Model-CRP/albumin, we retrieved from the multivariate logistic regression the predicted probability of 90-day mortality for each patient. These probabilities were plotted against age and SOFA score (Figure 4). After plotting, the best curve was fitted using the loess strategy for fitting smooth curves. In order to evaluate the usefulness of the model, we divided between survivors and non-survivors, depicting two curves. Thus, it was possible to compare the predicted probability of mortality between actually alive and dead patients, stratified per age and SOFA score. So, it was possible to show when the prediction of the models better separate between alive and dead patients. Figures 4A and 4B show the predicted mortality using a model that does not incorporate any biomarkers. Figures 4C and 4D show the predicted mortality of the Model-CRP/albumin. In general, the risk of mortality increased with age and severity of illness. Interestingly, the Model-CRP/albumin showed a higher distance of probability to death between survivors and non-survivors than the model without biomarkers in young patients and in those with less severe disease. For example, for patients with 20 years old the predicted risks of mortality retrieved from the model without biomarkers was 14.4% and 15.0% (mean difference: 0.6%), for observed alive and dead patients, respectively. From the Model-CRP/albumin, the predicted risks of mortality was 14.8% and 19.1% (mean difference: 4.3%), for observed alive and dead patients, respectively. However, for patients with 80 years old the Model-CRP/albumin did not increase the distance between predicted versus observed mortality risks (model without biomarker: 38.1% and 58.0% (mean difference: 19.9%), for observed alive and dead patients respectively; model CRP/albumin: 44.0% and 63.1% (mean difference: 19.1%), for observed alive and dead patients, respectively.

## Discussion

The main finding of our study is that markers of inflammation and of the severity of illness, specifically CRP and albumin levels at discharge, correlate with long-term prognosis (up to 90 days) after a sepsis episode. Of note, the discharge CRP/albumin ratio appeared to show higher accuracy than the discharge CRP alone as a predictor of mortality at 90 days. Furthermore, for critically ill septic patients, the model that included the CRP/albumin ratio was more calibrated to predict long-term mortality than the model that included discharge CRP levels.

In critical care, long-term prognosis after a critical illness is a pivotal topic of interest [6]. Indeed, short-term evaluation of mortality and morbidity after sepsis may be insufficient for epidemiologic and clinical studies. It appears that long-term prognosis is defined by a complex interplay among the patient's baseline status, severity of critical illness, residual organ dysfunction and residual inflammation after discharge [4], [6], [26].

CRP levels are typically measured in the ICU and are obtained daily in some centers [9]. The role of CRP levels as an inflammatory marker is well established [8]. However, the value of CRP for prognosis after ICU discharge is controversial [11], [14]–[16], [27]. Ho and coworkers found that CRP levels at discharge were associated with ICU readmission and in-hospital mortality in a typical ICU population [14]. We showed previously that reduction in CRP during the 24 hours prior to discharge was associated with reduced in-hospital mortality [11]. Nevertheless, a large prospective study suggested that CRP levels at discharge were not predictive of ICU readmission or short-term mortality [15]. The largest study that evaluated CRP for long-term prognosis suggested that both the maximum levels of CRP and the CRP level at discharge correlated with long-term prognosis after critical illness [13]. However, only 8.1% of the enrolled patients in this study were admitted owing to infection or sepsis [13]. Moreover, none of the above-mentioned studies focused exclusively on septic patients, a specific population in which inflammatory markers at discharge may be of particular relevance [7], [28]. Our analysis suggests that CRP levels at discharge may correlate with long-term outcome in septic patients.

Albumin levels were also associated with prognosis after critical illness. Although most studies have focused on albumin at admission and prognosis, Al-Subaie et al. showed that hypoalbuminemia at discharge correlated with ICU readmission and unexpected deaths [15]. Lee also suggested that albumin was an important prognostic marker for pneumonia [28]. Hypoalbuminemia is associated with inflammation, previous malnutrition or both [15], [29]. Nevertheless, albumin levels should be interpreted as a marker of severity [29] and could be a reliable indicator of frailty, a physiological condition characterized by low functional reserve, high susceptibility to stressors and unstable homeostasis [30], [31]. Merging albumin and CRP into a single index has been suggested previously [21], [32], [33]. Some authors employed a CRP/albumin ratio, whereas others evaluated CRP/prealbumin as a severity index [32], [33]. We hypothesized that an increased CRP/albumin ratio would imply a higher inflammatory status and could be more consistent than CRP alone. Therefore, our results indicate that markers of residual inflammation upon ICU discharge correlate with increased long-term mortality. Interestingly, use of this model with these biomarkers, as shown in Figure 4, could be of more value for young and less-severe patients. This finding deserves further exploration. Therefore, these biomarkers may be useful for identifying patients who are typically considered low risk and who show minor effects of strong predictors of a poor outcome (i.e., age and organ dysfunction), as has already been reported for cardiovascular diseases [34].

Based on the present analysis, a CRP/albumin ratio >2 showed the greatest sensitivity and specificity in predicting mortality at 90 days. In addition, a multivariate model that included the CRP/albumin ratio was more calibrated than a model that included CRP only together with classic predictors, such as age and chronic comorbidities. Calibration and discrimination are important issues when evaluating a predictive model [35]. Discrimination evaluates how well the model can separate between patients who die and those who survive. Calibration assesses the model's ability to provide predictions that are close to the average observed outcome [35]. Therefore, even though Model-CRP and Model-CRP/albumin showed similar AUCs, the calibration data showed that they were not equivalent. It should be highlighted that in our analysis, the prediction capability of CRP, albumin and the CRP/albumin ratio is modest (Table 3), which limits its use as a sole marker of outcomes. Other studies that have investigated the role of inflammatory markers on sepsis also yielded similar results [14], [27], [36]. In contrast, both the model that used CRP and the model that used the CRP/albumin ratio for analysis (Model-CRP and Model-CRP/albumin, respectively) showed better accuracy when predicting mortality (AUC >0.80).

Another interesting finding of our analysis is that late mortality after ICU discharge is still higher in patients with a high CRP/Albumin ratio (Figure 3). This fining is contrary to the common belief that residual inflammation would have more pronounced effects on early mortality after ICU. However, the literature on septic and mixed ICU patients indicates that the residual effects of critical illness may last up to 90 days after ICU discharge [37], [38]. Studies that have linked CRP at discharge and post-ICU events reported similar results, suggesting that inflammatory biomarkers may be better markers of long-term prognosis [36].

Consistent with two other studies that focused exclusively on post-ICU mortality of septic patients, our results provide support that age, chronic comorbidities and organ dysfunction at ICU discharge are important predictors of post-ICU mortality [3], [39]. The mortality rate of septic patients after discharge is similar to that of other typical ICU patients. The mortality rate was higher in this study compared with previous studies (22% vs. 4%–10%). In contrast to those studies, we analyzed patients with an ICU stay of at least 72 hours. As shown by Sakr *et* *al.* [3], the mortality rate after ICU discharge for septic patients increased after 3 days in the ICU, which suggests that patients remaining in the ICU for at least 72 hours are at an increased risk for mortality after discharge [3].

There are several limitations to our study. This study is unicentric and is thus subject to bias. Sixty-five patients were excluded from the analysis because of unit transfer, major missing data or loss during long-term follow up, which could alter the results. In addition, the cause of death was not recorded, which makes it difficult to adequately establish a link between inflammation at discharge and the cause of death. The use of blood products containing albumin, such as fresh frozen plasma, and the correction of albumin levels for fluid balance were also not recorded. We did not collect data on DNR orders and terminal disease status, which could have affected our results. Although the presence of comorbidities was included in the models, we did not specifically use a comorbidity score, such as the Charlson comorbidity index [40].

## Conclusions

Residual inflammation upon ICU discharge evaluated using the CRP/albumin ratio is an independent risk factor for mortality at 90 days in critically ill septic patients. The use of the CRP/albumin ratio as a long-term marker of prognosis provides more consistent data than the standard CRP values alone. The CRP/albumin ratio could be useful to guide further research in the field of residual inflammation at discharge and outcomes.
